# RETRACTION: Elevated Glutathione Peroxidase 2 Expression Promotes Cisplatin Resistance in Lung Adenocarcinoma

**DOI:** 10.1155/omcl/9757854

**Published:** 2026-03-31

**Authors:** Oxidative Medicine and Cellular Longevity

RETRACTION: H. Du, B. Chen, N.‐L. Jiao, Y.‐H. Liu, S.‐Y. Sun, and Y.‐W. Zhang, “Elevated Glutathione Peroxidase 2 Expression Promotes Cisplatin Resistance in Lung Adenocarcinoma,” *Oxidative Medicine and Cellular Longevity*, vol. 2020 (2020) https://doi.org/10.1155/2020/7370157.

The above article, published online on 6 March 2020 in Wiley Online Library (wileyonlinelibrary.com), has been retracted following the agreement of the journal’s Chief Editor, Jeannette Vasquez‐Vivar; and John Wiley & Sons Ltd.

The retraction has been agreed upon following an investigation of the concerns raised by *Actinopolyspora biskrensis* on PubPeer [1], which identified several concerns related to Figure 3c. More specifically, the ShNC‐GPX2 panel appears to overlap with Figure 4c of the publication [2] from a different author group, in which it represents a completely different tissue.

Further concerns were identified in Figure 1a regarding the similarity of the Cleaved‐caspase3 band in the Gpx2 arm of the A549 group to the Cyclin D1 band in the ShGpx2‐2 arm of the A549/DDP group.

As a result of the investigation, the data and conclusions of this article are considered unreliable.

The authors agree with this retraction.
